# Comparative Efficacy of Pharmacotherapy for Macular Edema Secondary to Retinal Vein Occlusion: A Network Meta-analysis

**DOI:** 10.3389/fphar.2021.752048

**Published:** 2021-12-08

**Authors:** Sheng Gao, Yun Zhang, Xun Li, Ge Ge, Jianan Duan, Chunyan Lei, Yue Zeng, Zhaolun Cai, Meixia Zhang

**Affiliations:** ^1^ Department of Ophthalmology, West China Hospital, Sichuan University, Chengdu, China; ^2^ Research Laboratory of Macular Disease, West China Hospital, Sichuan University, Chengdu, China; ^3^ Department of Gastrointestinal Surgery, West China Hospital, Sichuan University, Chengdu, China

**Keywords:** retinal vein occlusion (RVO), macular edema (ME), anti-VEGF (vascular endothelial growth factor) agents, dexamethasone intravitreal implant, retinal laser photocoagulation, efficacy and safety, network meta-analysis

## Abstract

**Purpose:** This network meta-analysis was conducted to obtain the relative effectiveness of different pharmacotherapy of macular edema secondary to retinal vein occlusion (RVO) by summarizing all available evidences.

**Methods:** PubMed, Embase, and Cochrane Library databases were searched for all relevant randomized controlled trials. The outcomes were estimated through a network meta-analysis, including the mean change in best-corrected visual acuity (BCVA) from baseline, the proportion of patients who gained ≥15 letters in BCVA from baseline, the mean change in central retinal thickness (CRT).

**Results:** We identified 15 randomized controlled trials (RCTs) involving 3,431 patients with RVO in our study. Different therapeutic regimens were compared including three anti-vascular endothelial growth factor (VEGF) agents (ranibizumab, bevacizumab, and aflibercept), ranibizumab with laser, dexamethasone intravitreal implant, and laser. For branch RVO, ranibizumab 0.5 mg monthly [weighted mean difference (WMD) = 11, 95% confidence intervals (CrI) 3.6 to 19], ranibizumab 0.5 mg 3 + pro re nata (WMD = 9.4, 95% CrI 0.43–18) is most effective in terms of changes of BCVA and 15 letters or more of BCVA improvement. For central RVO, three anti-VEGF regimens can improve visual acuity and there is no significant difference of efficacy among ranibizumab, bevacizumab and aflibercept (*p* > 0.05). Ranibizumab 0.5 mg monthly could achieve additional efficacy in CRT reduction in eyes with branch RVO or central RVO (WMD = -130, 95% CrI -400 to 140 or WMD = -280, 95% CrI -590 to 16)). Dexamethasone intravitreal implant (WMD = 1.7, 95% CrI -4.2 to 7.1 or WMD = 0.38, 95% CrI -9.8 to 8.8)) did not show a significant improvement in visual acuity at the end of 6 months follow-up in eyes with branch RVO or central RVO.

**Conclusion:** In summary, this network meta-analysis demonstrated several anti-VEGF agents had equivalent effects on mean visual acuity changes and anatomical recovery in 6 months in eyes with branch or central RVO. Only one injection of dexamethasone intravitreal implant in 6 months could not maintain the visual benefit. Patients and clinicians could choose pharmacotherapies with further consideration toward personal factors.

## 1 Introduction

Retinal vein occlusion (RVO) is the second most common retinal vascular disease which threatens visual acuity (VA) through macular edema and neovascularization. The general prevalence rate of RVO was approximately 0.52% in 2008 and its rate increased with age (Rogers et al., 2010). RVO is classified into the branch RVO (BRVO) and the central RVO (CRVO) according to the partial or complete occlusion caused by occlusive location. Several studies have confirmed the efficacy of pharmacotherapy for RVO secondary macular edema including anti-vascular endothelial growth factor (anti-VEGF) and corticosteroids intravitreal injection (Brown et al., 2011; Campochiaro et al., 2011). The published guidelines highlight several therapeutic strategies as recommendable treatment for patients with macular edema secondary to RVO(Schmidt-Erfurth et al., 2019; Flaxel et al., 2020). Several meta-analyses were performed on the therapies of RVO. However, it is still limited to an incomplete comparison of pharmacotherapy, or only one of the BRVO or CRVO has been analyzed (Ford et al., 2014; Regnier et al., 2015; Sermsiri et al., 2018). The network meta-analysis overcomes the limitation of traditional meta-analysis and a shortage of head-to-head trials (Rücker, 2012).

To address the knowledge gap, we have conducted a Bayesian network meta-analysis that included both direct and indirect comparisons simultaneously to obtain the comparative effectiveness of different pharmacotherapy of macular edema secondary RVO(Dias et al., 2013).

## 2 Methods

### 2.1 Protocol and Registration

The study protocol is registered in INPLASY (INPLASY202070012). The study was structured based on the PRISMA guidelines for Network Meta-analyses (Hutton et al., 2015). The protocol for this network meta-analysis had been published on Medicine (Zhang et al., 2020). The study aims to evaluate the efficacy and safety of intravitreal pharmacotherapies to obtain a comprehensive treatment recommendation for macular edema secondary to RVO.

### 2.2 Information Sources and Search Strategy

We systematically searched the electronic PubMed, Embase, and Cochrane Library databases (last updated on October 1, 2020). The detailed search strategies were presented in the Supplementary Table S1.

### 2.3 Eligibility Criteria

We summarized the detailed eligibility criteria according to the PICOS approach (patient, intervention, comparison, outcome, study design type) (Guyatt et al., 2011).

#### 2.3.1 Patients and Comparison of Interventions

The randomized controlled trials (RCTs) that compared two or more of the following treatment strategies (different anti-VEGF monotherapy regimens, anti-VEGF agent combined with laser photocoagulation, intravitreal corticosteroid monotherapy, and sham-controlled group (only the patients who received the sham injections for 6 months)) for patients with BRVO or CRVO were included in our analysis. We only analysed the agent dose that was approved or recommended by the guidelines to maximize the clinical significance for our study, including ranibizumab 0.5 mg, bevacizumab 1.25 mg, aflibercept 2 mg, conbercept 0.5 mg, dexamethasone intravitreal implant 0.7 mg, and triamcinolone acetonide 1 mg. Both laser photocoagulation and anti-VEGF combined with laser therapy were included in our analysis to provide more indirect data.

#### 2.3.2 Outcomes

Trials included should contain at least one of the outcomes in BRVO or CRVO. The outcomes included the mean change in BCVA from baseline (only the ETDRS results used for visual acuity were included in analysis), the proportion of patients who gained ≥15 letters in BCVA from baseline, and the mean change in CRT from baseline.

All the outcomes were analyzed at 6 months.

### 2.4 Study Selection and Data Collection

The studies were screened and selected independently by two reviewers and the relevant data were extracted from the included studies. The two reviewers (SG and YZ) summarized all study characteristics using the same standardized collection form. Any disagreement was resolved in discussion with another reviewer (CL) to reach a consensus.

### 2.5 Risk of Bias

The risk of bias of individual studies was assessed by the Cochrane Collaboration’s method. Studies were evaluated based on sequence generation, allocation concealment, blinding, selective reporting, incomplete outcome data, and other kinds of bias (Higgins et al., 2011). Disagreements were resolved by discussion with another reviewer (ZC) as an arbitrator to reach a consensus.

### 2.6 Data Synthesis and Statistical Analysis

The network meta-analyses were implemented within a Bayesian framework using Stata 14 (Stata Corp, College Station, TX, United States ), JAGS, and R (version x64 3.5.1). Random-effects models were used to evaluate the heterogeneity (Rücker and Schwarzer, 2015). The preferred outcome measures were reported as the relative risk (RR) with its 95% confidence intervals (CrI) and weighted mean difference (WMD) with its 95% CrI for dichotomous data and continuous data, respectively. To estimate the consistency between direct and indirect comparisons, we used the node-splitting method to calculate the inconsistency of the model. The inconsistency was reported by Bayesian *p*-value. A *p*-value < 0.05 indicated a significant inconsistency (Dias et al., 2010). We estimated the treatments for each outcome base on potential ranking probabilities which were calculated by the surface under the cumulative ranking curve (SUCRA) for each intervention (Salanti et al., 2011). The SUCRA value ranged from 0 to 1, the higher SUCRA value represented the better efficacy of treatment (Valk et al., 2009). To ensure the feasibility of our network meta-analysis, we drew the network plots to illustrate the comparisons of interventions across trials. Trials were excluded if the investigated treatment lacked the network connective nodes.

## 3 Results

### 3.1 Study Selection and Characteristics of Included Studies

**FIGURE 1:**
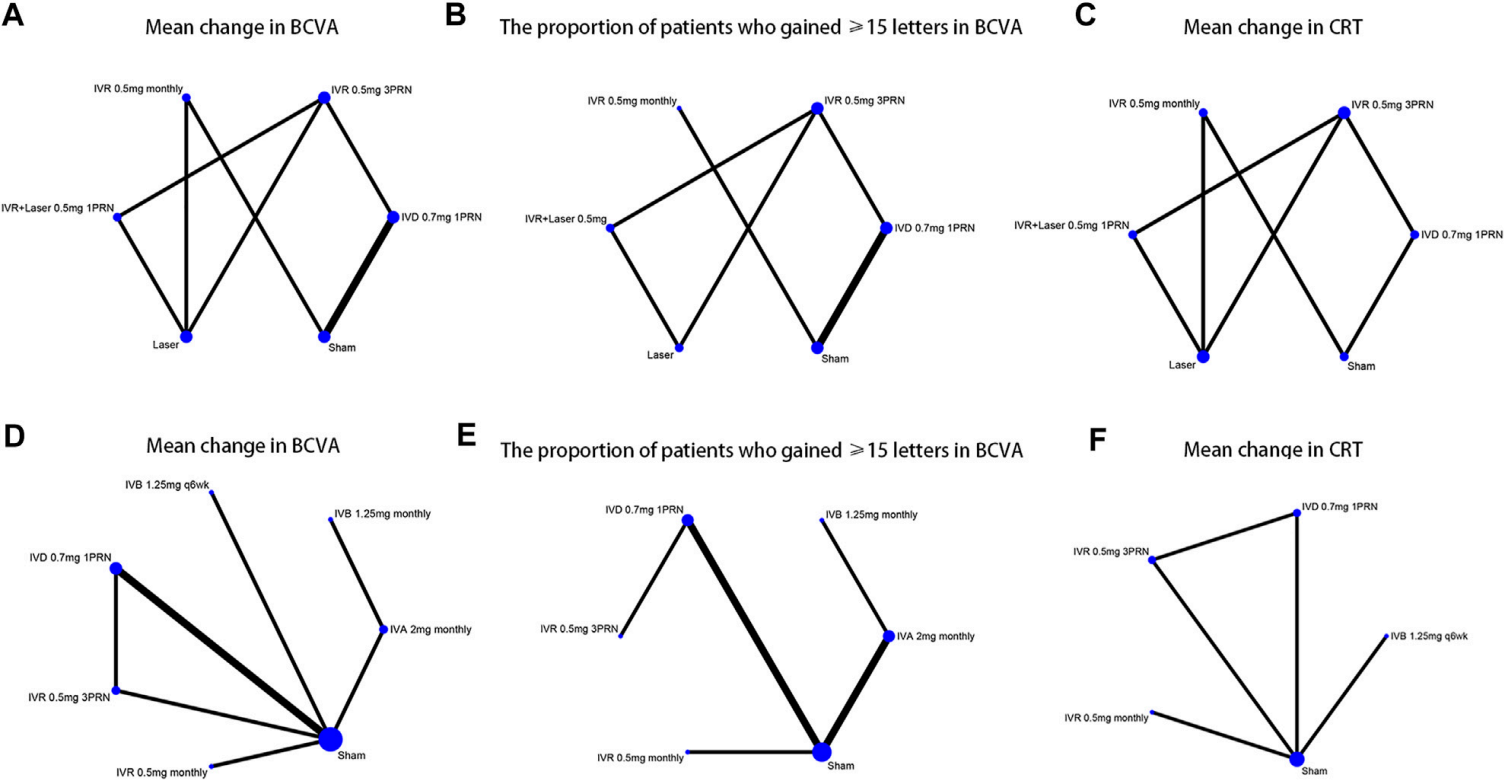
Network of the comparisons for the Bayesian network meta-analysis **(A–C)** The efficacy outcomes of macular edema secondary to branch retinal vein occlusion **(D–F)** The efficacy outcomes of macular edema secondary to central retinal vein occlusion.

We identified 1,044 potentially relevant studies. Fifteen RCTs that conformed to the inclusion criteria were contained in the network analysis, including six RCTs for BRVO(Haller et al., 2010; Campochiaro et al., 2011; Tan et al., 2014; Li X. et al., 2017; Tadayoni et al., 2017; Hattenbach et al., 2018) and nine RCTs for CRVO(Brown et al., 2010a; Haller et al., 2010; Kinge et al., 2010; Boyer et al., 2012; Epstein et al., 2012; Holz et al., 2013; Hoerauf et al., 2016; Scott et al., 2017a; Li X. et al., 2017). Overall, a total of 3,431 patients with macular edema secondary to RVO were involved in the study. The included trials compared the following eight interventions: intravitreal ranibizumab (IVR) 0.5 mg as needed after three initial monthly injections (3PRN); IVR 0.5 mg monthly; IVR with laser as soon as indicated by the investigators (IVR with the laser); dexamethasone intravitreal implant (DEX implant) 0.7 mg; intravitreal aflibercept (IVA) 2 mg monthly; intravitreal bevacizumab (IVB) 1.25 mg monthly; IVB 1.25 mg every 6 weeks (q6wk); laser therapy alone; and sham-controlled. Triamcinolone acetonide and conbercept were excluded for the absence of data and shortage of trials that connects the network nodes (Ramezani et al., 2012; Li F. et al., 2017; Sun et al., 2017). The network plots of all analytical comparisons are shown in Figure 1. The characteristics of the included trials are summarized in Table 1. The literature screening and selection process is shown in Supplementary Figure S1.

**TABLE 1:** Study and patient population characteristics of included studies.

Author, year	Treatment	Dose	Therapeutic regimen	Sample size	Mean age	Efficacy outcomes
BRVO						
Hattenbach, L. O., et al. 2018	IVR	0.5 mg	3PRN	126	NR	①②③
	IVD	0.7 mg	1	118		
Li, X., et al. 2017	IVD	0.7 mg	1	63	54.6	①②③
	Sham	—	—	65	53.0	
Tadayoni, R., et al. 2016	IVR	0.5 mg	3PRN	183	64.7	①②③
	IVR + laser	0.5 mg + laser	3PRN	180	67.3	
		—	—	92	67.7	
Tan, M. H., et al. 2014	IVR	0.5 mg	Monthly	15	69.6	①③
	Laser	—	—	21	66.7	
Haller, J. A., et al. 2010	IVD	0.7 mg	1	291	64.7	①②
	Sham	-	1	279	63.9	
Campochiaro, P. A., et al. 2011	IVR	0.5 mg	Monthly	131	67.5	①②③
	Sham	—	—	132	65.2	
CRVO						
Scott, I. U., et al. 2017	IVA	2 mg	Monthly	180	69	①②
	IVB	1.25 mg	Monthly	182	69	
Li, X., et al. 2017	IVD	0.7 mg	1	66	54.6	①②③
	Sham	—	—	65	53.0	
Hoerauf H, et al. 2016	IVR	0.5 mg	3PRN	124	65.3	①②③
	IVD	0.7 mg	1	119	66.9	
Holz, F. G., et al. 2013	IVA	2 mg	Monthly	106	59.9	①②
	Sham	-	Monthly	71	63.8	
Epstein, D. L., et al. 2012	IVB	1.25 mg	Q6w	30	70.6	①③
	Sham	-	-	30	70.4	
Boyer, D., et al. 2012	IVA	2 mg	Monthly	114	65.5	①②
	Sham	—	—	73	67.5	
Kinge, B., et al. 2010	IVR	0.5 mg	3PRN	16	72	①③
	Sham	—	—	16	72	
Haller, J. A., et al. 2010	IVD	0.7 mg	1	136	64.7	①②
	Sham	—	1	147	63.9	
Brown, D. M., et al. 2010	IVR	0.5 mg	Monthly	130	67.6	①②③
	Sham	—	—	130	65.4	

Efficacy outcome: ①Mean change in BCVA; ②The proportion of patients who gained ≥15 letters in BCVA from baseline; ③Mean change in CRT from baseline;

Abbreviations: BCVA, best-corrected visual acuity; BRVO, branch retinal vein occlusion; CRT, central retinal thickness; CRVO, central retinal vein occlusion; IVA, intravitreal aflibercept; IVB, intravitreal bevacizumab; IVD, intravitreal dexamethasone implant; IVR, intravitreal ranibizumab; NR, not reported; PRN, pro re nata; Q6w, every six weeks.

### 3.2 BRVO

#### 3.2.1 Mean Change in BCVA From Baseline

**FIGURE 2:**
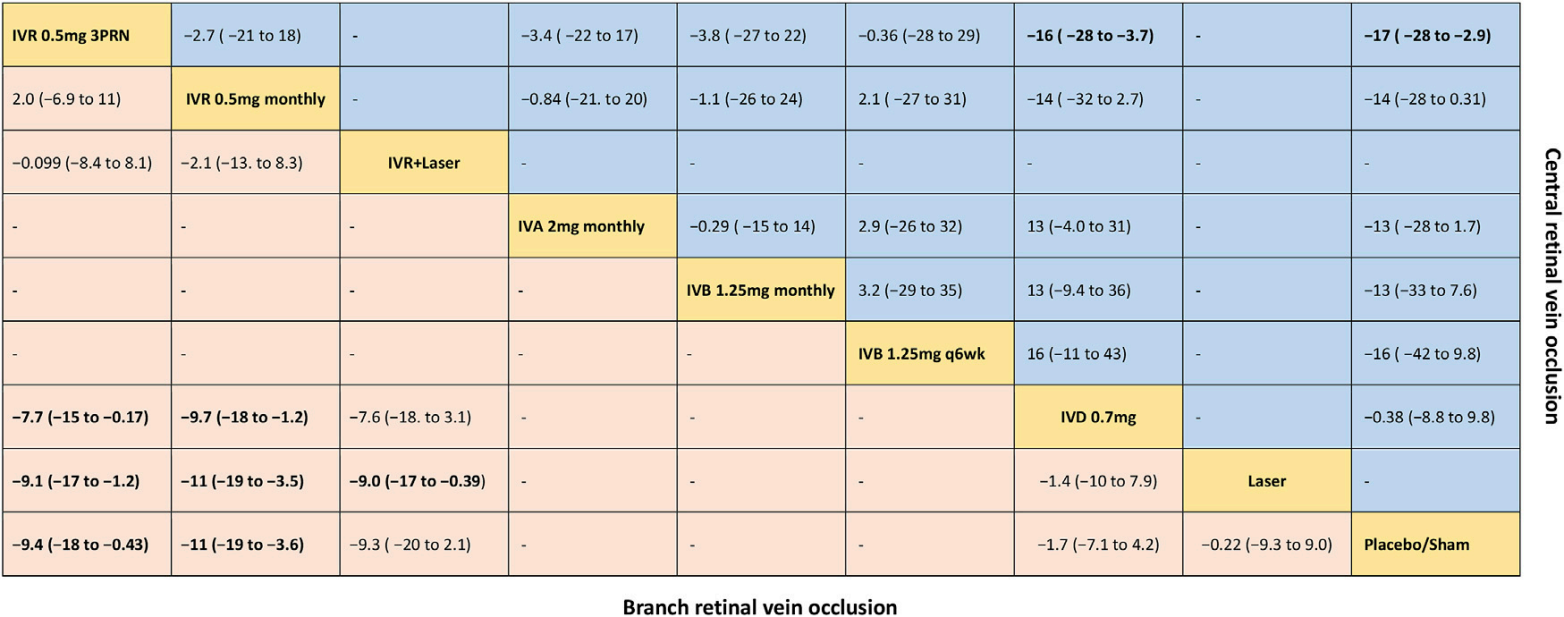
Comparative effectiveness of pharmacotherapies in terms of the mean change in BCVA for macular edema secondary to retinal vein occlusion in network meta-analysis. Weighted mean difference (95% credible interval) for comparisons are in cells in common between column-defining and row-defining treatment. Bold cells are significant. For branch retinal vein occlusion, weighted mean difference <0 favors row-defining treatment. For central retinal vein occlusion, weighted mean difference <0 favors column-defining treatment.

Six trials comparing six interventions in terms of mean change in BCVA at 6 months from baseline were examined (Haller et al., 2010; Campochiaro et al., 2011; Tan et al., 2014; Li X. et al., 2017; Tadayoni et al., 2017; Hattenbach et al., 2018). Figure 1A and Figure 2 showed separately the network plots and the results based on a Bayesian network meta-analysis that combines direct and indirect comparisons. Both IVR 0.5 mg monthly and 3PRN showed a statistically significant mean change in BCVA compared with sham-controlled. IVR with laser therapy showed a statistically nonsignificant trend toward meaningful change in BCVA compared with sham-controlled. However, both DEX implant, the laser alone, and sham-controlled were not superior to the other. The mean change in BCVA at 6 months from baseline, ordered from the most to least effective therapies based on the SUCRA values, were as follows: IVR 0.5 mg monthly [WMD = 11 with 95% CrI (3.6, 19), SUCRA = 88%], IVR 0.5 mg 3PRN [WMD = 9.4 with 95% CrI (0.43, 18), SUCRA = 74%], IVR with laser [WMD = 9.3 with 95% CrI (-2.1, 20), SUCRA = 73%], DEX implant 0.7 mg [WMD = 1.7 with 95% CrI (-4.2, 7.1), SUCRA = 31%], and laser alone therapy [WMD = 0.22 with 95% CrI: (-9.0, 9.3), SUCRA = 18%].

#### 3.2.2 The Proportion of Patients Who Gained ≥15 Letters in BCVA

**FIGURE 3:**
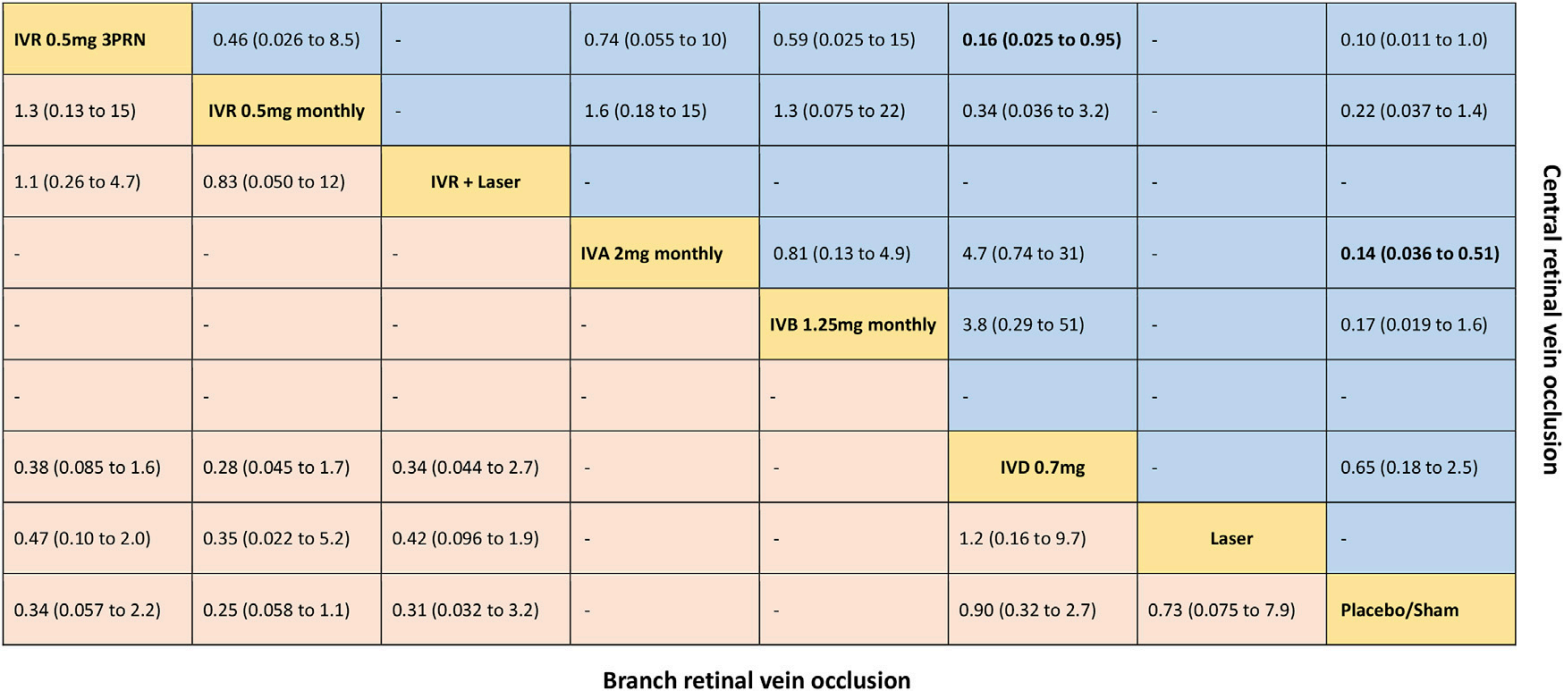
Comparative effectiveness of pharmacotherapies in terms of the proportion of patients who gained ≥15 letters in BCVA for macular edema secondary to retinal vein occlusion in network meta-analysis. Relative risk (95% credible interval) for comparisons are in cells in common between column-defining and row-defining treatment. Bold cells are significant. For branch retinal vein occlusion, relative risk <1 favors row-defining treatment. For central retinal vein occlusion, relative risk <1 favors column-defining treatment.

Five trials comparing six interventions contributed to the analysis of the proportion of patients who gained ≥15 letters in BCVA (Haller et al., 2010; Campochiaro et al., 2011; Tadayoni et al., 2016; Li X. et al., 2017; Hattenbach et al., 2018). Figure 1B and Figure 3 showed individually the network plots and the results of the network meta-analysis. Both IVR 0.5 mg monthly, IVR 0.5 mg 3PRN, and IVR with laser therapy showed a statistically nonsignificant trend toward improved the proportion of patients who gained ≥15 letters in BCVA. Both DEX implant, the laser alone, and sham-controlled were not clearly superior to the other. The proportion of patients who gained ≥15 letters in BCVA at 6 months from baseline, ordered from the most to least effective therapies based on the SUCRA values, were as follows: IVR 0.5 mg monthly [RR = 3.9 with 95% CrI (0.91, 17), SUCRA = 80%], IVR with laser [RR = 3.3 with 95% CrI (0.31, 31), SUCRA = 75%], IVR 0.5 mg 3PRN [RR = 3 with 95% CrI (0.46, 17), SUCRA = 70%], laser alone therapy [RR = 1.4 with 95% CrI (0.13, 13), SUCRA = 32%], and DEX implant 0.7 mg [RR = 1.1 with 95% CrI: (0.37, 3.1), SUCRA = 24%].

#### 3.2.3 Mean Change in Central Retinal Thickness From Baseline

**FIGURE 4:**
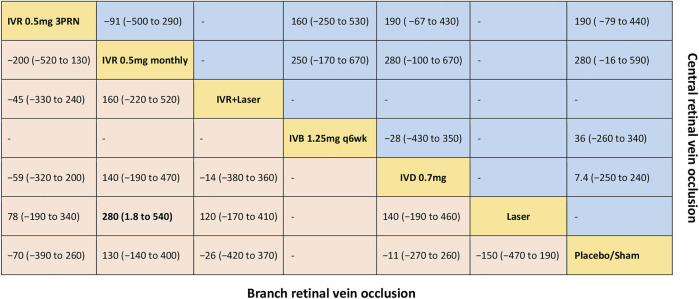
Comparative effectiveness of pharmacotherapies in term of the mean change in central retinal thickness for macular edema secondary to retinal vein occlusion in network meta-analysis. Weighted mean difference (95% credible interval) for comparisons are in cells in common between column-defining and row-defining treatment. Bold cells are significant. For branch retinal vein occlusion, weighted mean difference <0 favors row-defining treatment. For central retinal vein occlusion, weighted mean difference <0 favors column-defining treatment.

Five trials comparing six interventions in terms of mean change in CRT were evaluated (Campochiaro et al., 2011; Tan et al., 2014; Tadayoni et al., 2016; Li X. et al., 2017; Hattenbach et al., 2018). Figure 1C and Figure 4 showed separately the network plots and the results of the network meta-analysis. IVR 0.5 mg monthly showed a statistically nonsignificant trend toward improved central retinal thickness. Both IVR 0.5 mg 3PRN, IVR with laser therapy, DEX implant, the laser alone, and sham-controlled were not superior to the other. The mean change in central retinal thickness at 6 months from baseline, ordered from the most to least effective therapies based on the SUCRA values, were as follows: IVR 0.5 mg monthly [WMD = -130 with 95% CrI (-400, 140), SUCRA = 88%], DEX implant 0.7 mg [WMD = 11 with 95% CrI (-260, 270), SUCRA = 54%], IVR with laser [WMD = 26 with 95% CrI (-370, 420), SUCRA = 50%], IVR 0.5 mg 3PRN [WMD = 70 with 95% CrI (-260, 390), SUCRA = 36%], and laser alone therapy [WMD = 150 with 95% CrI: (-190, 470), SUCRA = 15%].

### 3.3 CRVO

#### 3.3.1 Mean Change in BCVA From Baseline

Nine trials comparing seven interventions in terms of mean change in BCVA at 6 months from baseline were evaluated (Brown et al., 2010a; Haller et al., 2010; Kinge et al., 2010; Boyer et al., 2012; Epstein et al., 2012; Holz et al., 2013; Hoerauf et al., 2016; Scott et al., 2017a; Li X. et al., 2017). Figure 1D and Figure 2 showed separately the network plots and the results of the network meta-analysis. The mean change in BCVA at 6 months from baseline, ordered from the most to least effective therapies based on the SUCRA values, were as follows: IVR 0.5 mg 3PRN [WMD = 17 with 95% CrI (2.9, 28), SUCRA = 76%], IVB 1.25 mg q6wk [WMD = 16 with 95% CrI (-9.8, 42), SUCRA = 67%], IVR 0.5 mg monthly [WMD = 14 with 95% CrI (-0.31, 28), SUCRA = 64%], IVA 2 mg monthly [WMD = 13 with 95% CrI (-1.7, 28), SUCRA = 60%], IVB 1.25 mg monthly [WMD = 13 with 95% CrI (-7.6, 33), SUCRA = 58%], DEX implant 0.7 mg [WMD = 0.38 with 95% CrI: (-9.8, 8.8), SUCRA = 14%].

#### 3.3.2 The Proportion of Patients Who Gained ≥15 Letters in BCVA

Seven trials comparing six interventions contributed to the analysis of the proportion of patients who gained ≥15 letters in BCVA (Brown et al., 2010a; Haller et al., 2010; Boyer et al., 2012; Holz et al., 2013; Hoerauf et al., 2016; Scott et al., 2017a; Li X. et al., 2017). Figure 1E and Figure 3 showed separately the network plots and the results of the network meta-analysis. IVA 2 mg monthly showed a statistically significant gained ≥15 letters in BCVA compared with sham-controlled. The proportion of patients who gained ≥15 letters in BCVA at 6 months from baseline, ordered from the most to least effective therapies based on the SUCRA values, were as follows: IVR 0.5 mg 3PRN [RR = 9.8 with 95% CrI (1.0, 89), SUCRA = 82%], IVA 2 mg monthly [RR = 7.2 with 95% CrI (2.0, 28), SUCRA = 75%], IVB 1.25 mg monthly [RR = 5.8 with 95% CrI (0.62, 53), SUCRA = 62%], IVR 0.5 mg monthly [RR = 4.6 with 95% CrI (0.74, 27), SUCRA = 54%], DEX implant 0.7 mg [RR = 1.5 with 95% CrI: (0.40, 5.7), SUCRA = 21%].

#### 3.3.3 Mean Change in Central Retinal Thickness From Baseline

Five trials comparing five interventions in terms of mean change in CRT were examined (Brown et al., 2010a; Kinge et al., 2010; Epstein et al., 2012; Hoerauf et al., 2016; Li X. et al., 2017). Figure 1F and Figure 4 shows separately the network plots and the results of the network meta-analysis. The mean change in central retinal thickness at 6 months from baseline, ordered from the most to least effective therapies based on the SUCRA values, were as follows: IVR 0.5 mg monthly [WMD = -280 with 95% CrI (-590, 16), SUCRA = 91%], IVR 0.5 mg 3PRN [WMD = -190 with 95% CrI (-440, 79), SUCRA = 74%], IVB 1.25 mg monthly [WMD = -36 with 95% CrI (-340, 260), SUCRA = 38%], DEX implant 0.7 mg [WMD = -7.4 with 95% CrI: (-240, 250), SUCRA = 26%].

### 3.4 Quality of Evidence

**FIGURE 5:**
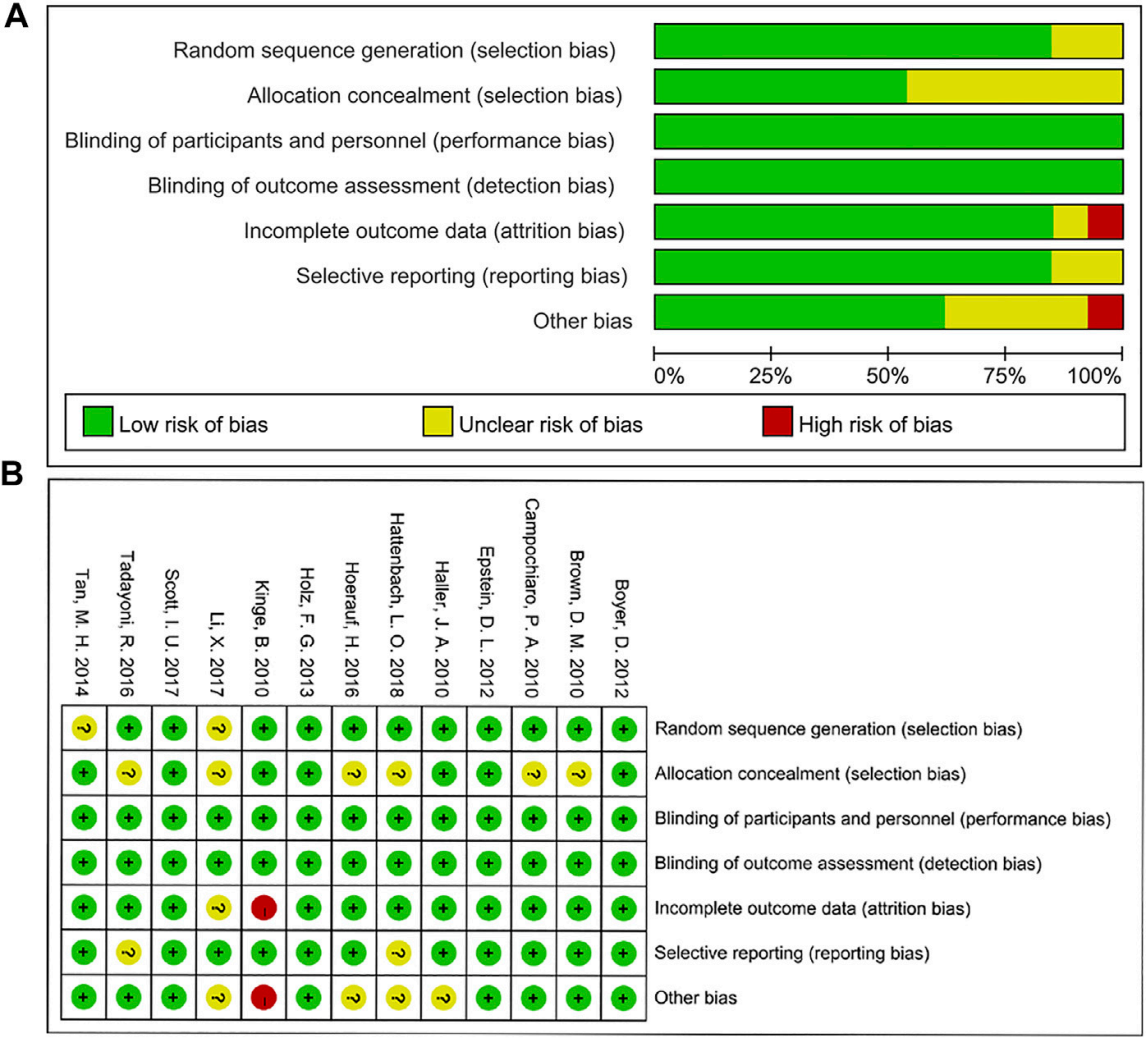
Risk of bias graph **(A)** and summary **(B)**.

The bias assessment for eligible RCTs included in the network meta-analysis is shown in Figure 5 according to the Cochrane risk-of -bias tool, suggesting no severe risk of bias. The results of node-splitting analysis and their *p*-value were larger than 0.05 which demonstrated no statistical inconsistency between direct and indirect comparisons among all outcomes in any closed loops.

## 4 Discussion

In the network meta-analysis, we compared the efficacy of different pharmacotherapies for BRVO or CRVO comprehensively. Anti-VEGF agents can improve visual acuity and recover retinal anatomical structure in patients with both BRVO and CRVO at 6 months. DEX implant and laser alone did not show a significant improvement in visual acuity at the end of 6 months follow-up both in BRVO and CRVO. For BRVO, anti-VEGF combined with laser therapy showed no statistically significant difference in improving vision or reducing CRT at 6 months compared with anti-VEGF monotherapy. In general, our result confirmed the results of head-to-head RCTs including the VIBRANT, BRIGHTER, RABAMES, GALILEO, CRUISE, and COPERNICUS trials, which was expanded and consistent with the previous meta-analysis (Brown et al., 2010b; Boyer et al., 2012; Holz et al., 2013; Campochiaro et al., 2015; Pielen et al., 2015; Tadayoni et al., 2016).

In the analysis of the mean change of BCVA and visual benefits, all the anti-VEGF agents with different therapeutic regimens within our evaluation system showed better clinical benefit for visual acuity at 6 months. Patients and clinicians could make decisions in conjunction with other factors, such as personal preference, cost, the intravitreal injection frequency, and follow-up burden (Justis et al., 2017). It is worth noting that the baseline VA is the important predictor for final VA like the final vision was lower in those with poor baseline BCVA even with a relatively higher mean change of BCVA (Boyer et al., 2007; Gupta, 2008; Bressler and Susan, 2012). The baseline characteristics of baseline VA are similar between the CRUISE study and COMRADE-C study, while the time between diagnosis and randomization is longer in the CRUISE study (3.3 months) than in the COMRADE-C study (about 1.5 months) (Brown et al., 2010b; Hoerauf et al., 2016). At the end of 6 months follow-up, even if the injection number is more in CRUISE study with a monthly therapeutic regimen, the mean change of BCVA is lower in the CRUISE study (14.9 letters) compared with the COMRADE-C study (16.9 letters), like the proportion of patients gained ≥15 letters are 47.7 versus 58.9%.

In terms of reducing CRT and recovering the retinal anatomical structure, except for the IVR 0.5 mg monthly in BRVO and 0.5 mg monthly or 3PRN in CRVO showed a statistically nonsignificant trend toward decreased CRT, the other treatments were not clearly superior to the sham-controlled group at month 6. This might be related to the natural course of disorders that macular edema might persist or resolve itself over time (Scott et al., 2017b). The CRT represented a rapid decline then relative stability with anti-VEGF agents. When using DEX implant, it showed a rapid decline to bottom around month two then reoccurring increase without retreatment (Haller et al., 2010; Li X. et al., 2017). As the absence of data about aflibercept and the shortage of trails that connect the network nodes, the trials investigating the CRT decline in aflibercept were excluded (Boyer et al., 2012; Holz et al., 2013). In the SCORE2 study, although there was no statistically significant between aflibercept and bevacizumab in mean change of CRT, the complete resolution of fluid was significantly higher in the aflibercept group compared with the bevacizumab group in post hoc analyses (Scott et al., 2017a). The effectiveness of aflibercept on functional and anatomic outcomes deserves attention.

For DEX implant both in BRVO and CRVO, the results of trials we included were consistent, and all DEX implant groups received a single DEX implant injection followed by sham injections in 6 months (Haller et al., 2010; Hoerauf et al., 2016; Li X. et al., 2017). In the GENEVA study, the mean BCVA achieved an apex of about 10 letters at month two in all RVO, while this value decreased progressively and reached approximately 7 letters in BRVO and baseline level in CRVO at month 6. As well as the proportion of patients who gained ≥15 letters, it was significantly greater in DEX implant groups than a sham group in the first 3 months, but the difference between two groups was no longer statistically significant at month 6 both in BRVO and CRVO(Haller et al., 2010). Similar variation trends of BCVA and visual benefits were observed in the COMRADE-C study and Li, X.‘s study (Hoerauf et al., 2016; Li X. et al., 2017). Hence, the BCVA improvements brought by a single DEX implant approximately continue for 3 months. The subsequently persistent decline suggests that patients need extra treatment within 6 months. The other study which gave another injection of DEX implant when BCVA decreased and macular edema increased around month four showed no significant difference between DEX implant and bevacizumab in mean change of BCVA and CRT at the end of 6 months follow-up (Gado and Macky, 2014). Therefore, additional RCTs of DEX implant with a shorter retreatment period would be needed to assess the efficacy of DEX implant, which might relatively reduce the advantage of anti-VEGF agents in our network meta-analysis.

The value of laser photocoagulation alone therapy for macular edema secondary to BRVO remains evaluated. Our network meta-analysis showed there is no significant difference between the laser group and sham-controlled group both in vision and anatomic outcomes. Meanwhile, the effectiveness of laser combined with ranibizumab is not superior to the anti-VEGF monotherapy. In the 6 months results of the BRIGHTER study, the number of anti-VEGF injections was 4.5 ± 1.2 in a combined group which was similar to the ranibizumab monotherapy (4.8 ± 1.0 injections) (Tadayoni et al., 2016). Prolonging to 24 months study, the mean number of ranibizumab injections was no different in combined arm and ranibizumab monotherapy either. The addition of laser did not obtain better functional outcomes or less treatment (Tadayoni et al., 2017).

The safety analysis was not included in our work for network connection failure caused by the absence of data. In general, the anti-VEGF agents both ranibizumab, bevacizumab, and aflibercept have a low incidence of increased intraocular pressure (IOP) and cataract. The adverse events and serious adverse events were no new safety events and were consistent with those reported in previous studies of age-related macular degeneration (Rosenfeld et al., 2006; Brown et al., 2009; Heier et al., 2012; Tadayoni et al., 2016). In terms of DEX implant, the treatment-related IOP increase is a well-known risk of intravitreal corticosteroid therapy (Yannuzzi et al., 2014; Aref et al., 2015). Ocular hypertension occurred significantly more frequently in the DEX implant group. The changes in IOP peaked around month two and declined progressively with no statistical difference from sham-controlled at month 6. The overall incidence of ocular adverse events was significantly higher in the DEX implant group. But the occurrence of cataracts and serious adverse events were no significant between DEX implant and sham-controlled group (Haller et al., 2010; Hoerauf et al., 2016).

In the analysis of the number of injections, there is no statistical significance between monthly injection and PRN regimen as similar functional outcomes and anatomical outcomes. It suggested that an individualized PRN regimen could reduce the treatment need and treatment burden both cost and follow-up monitoring. The treat-and-extend regimen was excluded for a shortage of trials that connects the network nodes. A recent RCT showed a significantly less number of injections with IVA T&E regimen compared with IVR T&E regimen, and no difference between two groups regarding vision and CRT(Casselholm De Salles et al., 2019). Although the DEX implant gradually released the drug over several months, the 6 months retreatment period seems too long to keep the vision and retinal structure. The studies of optimal retreatment period still need to be verified.

Several limitations in our present work merit further discussion. The limitations of the difficulty of investigations of potential heterogenicity, such as regional, ethnic, economic, and medical differences, were caused by the meta-analysis of aggregate data rather than individual patient data. Due to the obvious influence of initial VA and duration of disease on final vision, although the inclusion criteria were basic matching, they might also have a certain impact on our meta-analysis. Owing to the absence of data and shortage of trials that connects the network nodes, the trials including aflibercept in BRVO, triamcinolone acetonide, and conbercept were excluded from our work, which causes the types of pharmacotherapies included in our work less than the actual agents available in the clinic.

Despite the above-mentioned limitations, our study has several strengths. To the best of our knowledge, this is the first network meta-analysis to quantitatively review the effectiveness of anti-VEGF therapy and DEX implant for BRVO and CRVO comprehensively. Second, we had strict inclusion criteria and separated all the different therapeutic regimens to avoid potential differences caused by individual clinical intervals. Third, the lack of statistically significant inconsistency in our work confirms the accuracy of the results.

In conclusion, our results show that multiple pharmacotherapies would be effective treatments for macular edema secondary to RVO. Three anti-VEGF agents cause significant VA improvement and have equivalent effects on mean VA changes, vision benefits, and anatomical outcomes. In particular, ranibizumab 0.5 mg monthly shows relatively excellent performance. Only one injection of dexamethasone intravitreal implant in 6 months could not maintain the visual benefit, but it might improve the speed and incidence of visual improvement in the short term. While the ocular adverse events and optimal long-term dosing schedule still need attentions. Patients and clinicians could choose drugs with further consideration toward personal factors such as patient preference, individual treatment response, convenience of dosing, financial constraints, and evolving regulatory standards.

## Data Availability

The original contributions presented in the study are included in the article/Supplementary Material further inquiries can be directed to the corresponding authors.
