# Supplementary material for: Comparative Efficacy of Pharmacotherapy for Macular Edema Secondary to Retinal Vein Occlusion: A Network Meta-analysis
Source: Front Pharmacol. 2021 Dec 8;12:752048. doi: 10.3389/fphar.2021.752048 (PMC8692786; doi:10.3389/fphar.2021.752048)

Supplementary Material

# Table S1. Literature Search Strategy (October 1, 2020)

| PubMed | Embase | Cochrane library |
| --- | --- | --- |
| #1 (((((retinal vein occlusion) OR "Retinal Vein Thromboses") OR "Retinal Vein Thrombosis") OR "Retinal Vein Occlusions"))  #2 ((((macular edema) OR "Cystoid Macular Edema")) OR "macular oedema")  #3 #1 AND #2 | #1 retinal vein occlusion.mp. or retina vein occlusion/  #2 Retinal Vein Thromboses.mp.  #3 Retinal Vein Thrombosis.mp.  #4 Retinal Vein Occlusions.mp.  #5 1 or 2 or 3 or 4  #6 macular edema.mp. or retina macula edema/ or macular edema/ or retina macula cystoid edema/  #7 Cystoid Macular Edema.mp.  #8 macular oedema.mp.  #9 6 or 7 or 8  #10 5 and 9  #11 random:.tw. or placebo:.mp. or double-blind:.tw.  #12 10 and 11 | #1 MeSH descriptor: [Retinal Vein Occlusion] explode all trees  #2 ("Retinal Vein Thromboses"):ti,ab,kw  #3 ("Retinal Vein Thrombosis"):ti,ab,kw  #4 ("Retinal Vein Occlusions"):ti,ab,kw  #5 ("retinal vein occlusion"):ti,ab,kw  #6 #1 OR #2 OR #3 OR #4 OR #5  #7 MeSH descriptor: [Macular Edema] explode all trees  #8 ("Macular Edema"):ti,ab,kw  #9 ("Cystoid Macular Edema"):ti,ab,kw  #10 ("macular oedema"):ti,ab,kw  #11 #7 OR #8 OR #9 OR #10  #12 #6 AND #11 |

# Figure S1. PRISMA flow diagram of eligible studies.


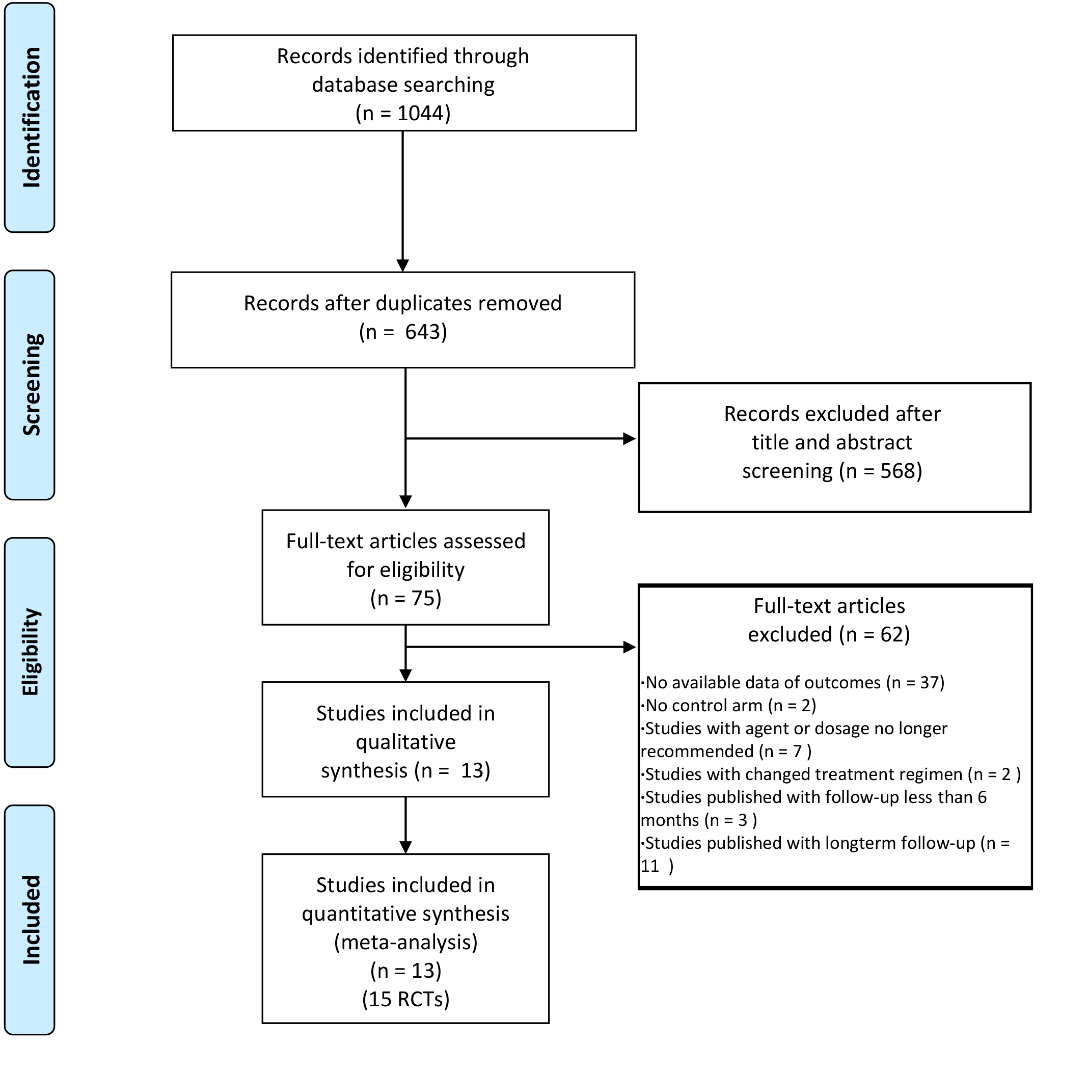

Supplement: Supplementary file 1 [file DataSheet1.docx]
